# Per-protocol analysis of the ZINC trial for HIV disease among alcohol users

**DOI:** 10.1186/s13063-021-05178-9

**Published:** 2021-03-23

**Authors:** Sara Lodi, Matthew Freiberg, Natalia Gnatienko, Elena Blokhina, Tatiana Yaroslavtseva, Evgeny Krupitsky, Eleanor Murray, Jeffrey H. Samet, Debbie M. Cheng

**Affiliations:** 1 Department of Biostatistics, Boston University School of Public Health, 801 Massachusetts Avenue, Boston, MA 02118 USA; 2 Vanderbilt Center for Clinical Cardiovascular Trials Evaluation (V-C3REATE), Cardiovascular Division, Vanderbilt University Medical Center, Nashville, TN USA; 3 Department of Medicine, Section of General Internal Medicine, Boston Medical Center, Clinical Addiction Research and Education (CARE) Unit, Boston, MA USA; 4 First Pavlov State Medical University of St. Petersburg, St. Petersburg, Russian Federation; 5 Department of Addictions, V.M. Bekhterev National Medical Research Center for Psychiatry and Neurology, St. Petersburg, Russian Federation; 6 Department of Epidemiology, Boston University School of Public Health, Boston, MA USA; 7 Department of Medicine, Section of General Internal Medicine, Boston Medical Center, Clinical Addiction Research and Education (CARE) Unit, Boston University School of Medicine, Boston, MA USA; 8 Department of Community Health Sciences, Boston University School of Public Health, Boston, MA USA

**Keywords:** Zinc, Alcohol abuse, HIV, Per-protocol, Inverse probability weighting

## Abstract

**Background:**

The Zinc for INflammation and Chronic disease in HIV (ZINC) trial randomized person who live with HIV (PLWH) who engage in heavy drinking to either daily zinc supplementation or placebo. The primary outcome was change in the Veterans Aging Cohort Study (VACS) index, a predictor of mortality, between baseline and 18 months. Because adherence and follow-up were suboptimal, the intention-to-treat analysis, which was not statistically significant, may have underestimated the effect of the zinc supplementation.

**Objective:**

We estimated the per-protocol effect of zinc versus placebo in the ZINC trial (i.e., the effect that would have been observed if all participants had had high adherence and none was lost to follow-up).

**Methods:**

Adherence was measured as the self-reported percentage of pills taken in the previous 6 weeks and assessed at all post-baseline visits. We used inverse probability weighting to estimate and compare the change in the VACS index at 18 months in the zinc and placebo groups, had all the trial participants had high adherence (i.e., cumulative adherence ≥80% at 18 months). To examine trends by level of adherence, we rerun the analyses using thresholds for high adherence of 70% and 90% of average self-reported pill coverage.

**Results:**

The estimated (95% confidence interval) change in the VACS index was − 2.16 (− 8.07, 3.59) and 5.84 (0.73, 11.80) under high adherence and no loss to follow-up in the zinc and placebo groups, respectively. The per-protocol effect estimate of the mean difference in the change between the zinc and placebo groups was − 8.01 (− 16.42, 0.01), somewhat larger than the intention-to-treat effect difference in change (− 4.68 (− 9.62, 0.25)), but it was still not statistically significant. The mean difference in the change between individuals in the zinc and placebo groups was − 4.07 (− 11.5, 2.75) and −12.34 (− 20.14, −4.14) for high adherence defined as 70% and 90% of pill coverage, respectively.

**Conclusions:**

Overall, high adherence to zinc was associated with a lower VACS score, but confidence intervals were wide and crossed 0. Further studies with a larger sample size are needed to quantify the benefits of zinc supplementation in this population.

**Trial registration:**

ClinicalTrials.gov NCT01934803. Registered on August 30, 2013

## Introduction

Alcohol use and inflammation are common among persons living with HIV (PLWH) and are linked to increased mortality and non-infectious disease morbidity. Zinc supplementation is associated with reduced inflammation in uninfected people and delayed immunologic failure among PLWH [1, 2]. The recent Zinc for INflammation and Chronic disease in HIV (ZINC) trial was a randomized placebo-controlled trial of daily zinc supplementation versus placebo among PLWH who engage in heavy drinking in Russia [3, 4]. The primary outcome was change in the Veterans Aging Cohort Study (VACS) index at 18 months after randomization. The VACS index is a prognostic score for PLWH, with higher values indicating a higher risk of mortality [5]. Components of the VACS index include age, CD4 cell count, HIV-1 RNA level, hemoglobin, FIB4 score [6], estimated glomerular filtration rate (EGFR), and hepatitis C co-infection status. In the intention-to-treat analysis of the ZINC trial, the VACS index increased between baseline and 18 months in both the zinc and placebo groups, indicating that both groups’ mortality risk had increased over the study period. However, the increase in VACS index was less pronounced in the zinc group than in the placebo group, even though not statistically significant, with a mean difference in change of − 4.68 points for zinc versus placebo (95% confidence interval [CI] − 9.62, 0.25; *p* = 0.06).

Despite every effort (reminder calls, text messaging, and compensation for attendance at study visits) to retain the participants in the study, adherence to the assigned medications was suboptimal (self-reported pill coverage < 80%) in 32% of the adherence assessments. Also, 54% of participants missed at least one study visit. Consequently, the intention-to-treat approach might have underestimated the efficacy of zinc [7]. A secondary per-protocol analysis was conducted restricting to participants who remained adherent during the study (i.e., with self-reported pill coverage ≥80% for at least three study visits) [4]. The estimated mean difference in change for zinc versus placebo in this sensitivity analysis was − 7.49 (− 13.74, − 1.23). However, like all per-protocol analyses based on standard methods, this analysis could have been subject to bias because the participants who chose to adhere to their treatment might have been systematically different from those who were non-adherent, i.e., with regard to their baseline and post-randomization prognostic factors [8]. Recently, novel methods for causal inference, called g-methods, have been used to estimate the per-protocol effect in randomized controlled trials [9–13]. These methods include inverse probability weighting, the parametric g-formula, and the g-estimation and estimate the expected value of the outcome under pre-specified levels of adherence or protocol compliance. Adherence is regarded as a time-varying post-randomization exposure variable whose effect on the outcome can be biased by baseline and time-varying confounders. Unlike the conventional methods, g-methods can adjust treatment effect estimates for baseline and post-randomization prognostic factors that are affected by treatment assignment. Like conventional methods, g-methods rely on the untestable assumptions of correct model specification and no unmeasured confounding between post-randomization treatment and the outcome.

Our main goal in this manuscript is to estimate the per-protocol effect in the ZINC trial using inverse probability weighting to minimize bias. This will complement the published intention-to-treat effect estimates by estimating the effect of daily zinc supplementation had all participants in the trial adhered to their medications and, unless they died during follow-up, had remained under follow-up for the duration of the study.

## Material and methods

### Study population

The protocol and study design of the ZINC trial was published elsewhere [3]. Briefly, participants were recruited between 2013 and 2015 from HIV and addiction care centers in St. Petersburg, Russia. The main inclusion criteria were documented HIV-positive status and self-reported heavy alcohol consumption in the past 30 days based on the National Institute of Alcohol Abuse and Alcoholism definition of risky drinking [14]. Eligible patients were randomized 1:1 to receive daily zinc supplementation or placebo to be taken daily for 18 months. The study was double-blinded as neither the participants nor the study research staff were aware of the randomization group. Study visits occurred at months 6, 12, and 18. At each study visit, all participants completed a questionnaire assessing drinking, depression, and injection drug use in the previous month; urine and blood samples were also collected. Study medications could be refilled at each study visit and at additional interim visits occurring at 6 weeks and at 3, 9, and 15 months (Additional file 1: Figure 1). Adherence was measured at each post-baseline visit in two ways: (1) self-reported percentage of pills taken in the past 6 weeks on the visual analog scale and (2) detection of riboflavin in a urine sample. Riboflavin was added to both zinc and placebo capsules and was detected in a room with low ambient light, using ultraviolet light at the long-wave setting. A positive riboflavin test was indicative of uptake of the study medications in the past 24 h. Reasons for low medication uptake were collected at each refill visit. The study included 254 individuals (126 and 128 in the zinc and placebo group, respectively). The distribution of socio-demographic characteristics and baseline prognostic factors was similar in the zinc and placebo groups [3, 4]. There were no serious adverse events that were related to the study mediation, and 27 participants (11 in the zinc group and 16 in the placebo group) reported minimal adverse effects with gastrointestinal events being the most common. Death occurred among 33 (13%) participants (21 in the zinc group and 11 in the placebo group) during follow-up, and there was no statistically significant difference in mortality by treatment group (*p* = 0.10). The cause of death by treatment group was reported in the main paper [3, 4]. Secondary outcomes, which were not examined in this per-protocol analysis, were (1) Reynolds risk score at 18 months, a marker for cardiovascular disease risk; (2) change in CD4 cell count between baseline and 18 months; and (3) biomarkers including interleukin 6, d-dimer, sCD14, intestinal fatty acid-binding protein (I-FABP), and lipopolysaccharide-binding protein (LBP) at 18 months. Written informed consent was obtained for all study participants. The study was approved by the Institutional Review Board of Boston University Medical Campus and of the First St. Petersburg Pavlov State Medical University.

For this per-protocol analysis, 2 of the 254 participants were excluded due to missing baseline VACS index and 4 more due to post-baseline HIV-negative status. This resulted in a cohort of 248 individuals (122 and 126 in the zinc and placebo groups, respectively). These exclusions did not occur in the intention-to-treat analysis, which relied on the random allocation of the individuals to zinc or placebo. For the present analyses, for each individual, follow-up started at the date of randomization and ended at the end of the study at 18 months, death, or loss to follow-up. The latter was defined as the first missed study visit. This definition of loss of follow-up was unnecessary in the intention-to-treat analysis, because it did not rely on post-randomization predictors of adherence. Instead, loss to follow-up in the intention-to-treat analysis was defined by the availability of information at the 18-month visit.

### Definition of adherence

Estimating the per-protocol effect requires a precise definition of what constitutes high adherence. As this was not reported in the study protocol, for the purpose of these analyses, high adherence at 18 months was defined as the average (cumulative) self-reported adherence ≥80% to the assigned medications (zinc supplementation or placebo tablets). As individuals who stop therapy due to clinical reasons related to the study medications are not deviating from the protocol, any treatment discontinuation due to serious adverse effects was labeled as high adherence. Because the riboflavin test could only detect medication uptake up to 24 h post-ingestion and self-reported adherence captured pill uptake over 6 weeks, for the purpose of this study, we used the latter as the primary adherence measure. When adherence was not measured at a refill visit, we carried forward the measurement of adherence from the most recent previous visit up to the next study visit. Participants refilled their study medications at all visits, and they received an extra 6-week supply to accommodate lost medications and missed visits.

### Statistical analyses

In the original intention-to-treat analysis, a linear regression model was fit to estimate the difference in change in VACS index adjusting for the randomization stratification factors (sex and heavy drinking in the 7 days prior to baseline). Multiple imputation using the iterative Markov chain Monte Carlo technique was performed to account for 95 missing VACS index measurements at 18 months.

To estimate the per-protocol effect adjusting for baseline and post-baseline factors, we used the following 2-step approach. In the first step, separately for each treatment group, we estimated the effect of high versus low cumulative adherence on change in VACS index at 18 months via a linear regression model. Because of the blinded nature of the trial, we expect no effect of adherence on the VACS index in the placebo group, and any effect of adherence would be indicative of residual confounding. However, if treatment were beneficial, we expect to observe an effect of adherence on the VACS index in the zinc group. The model included an indicator for high versus low adherence. To improve comparability with the intention-to-treat analysis, we also adjusted for sex and heavy drinking in the 7 days prior to baseline, the two randomization stratification factors. Death was considered a censoring event. In the second step, we obtained an estimate of the per-protocol effect by comparing the predicted mean change in VACS index in the zinc group versus the placebo group under high adherence.

To adjust for potential confounding, we weighted each participant by the inverse probability of having their own observed history of adherence [15–17]. To estimate the weights, we fit two separate pooled logistic regression models (one for each randomization group) for the probability of high adherence at each post-baseline visit. These models included age, sex, employment status, depressive symptoms, VACS score, past 30-day injection drug use, and past 7-day heavy drinking at baseline as well as the following post-randomization covariates measured at the previous most recent visit: indicators for self-reported use of antiretroviral treatment (ART), past 30-day injection drug use, past 30-day heavy drinking, depressive symptoms defined as Center for Epidemiological Study Depression (CES-D) score ≥ 16, CD4 cell count on the natural logarithm scale, VACS index, and adherence to study medications. These potential confounders were chosen a priori as potential common causes of poor adherence and mortality based on the literature and clinical knowledge.

To adjust for potential selection bias due to informative loss to follow-up (i.e., missing a study visit), we weighted each individual by the inverse probability of remaining uncensored up to the end of the study using a similar approach to the weighting for adherence. The two sets of weights were multiplied and stabilized [16]. Because adherence and loss to follow-up might be affected by treatment assignment at randomization, the models for the weights were fit separately in each randomization group. The mean of the stabilized weights was 0.99 (min 0.21, max 3.37). We used a non-parametric bootstrap procedure based on 500 samples to obtain percentile-based 95% confidence intervals (CIs). A step-by-step description of the estimation for the per-protocol effect is in Additional file 1: Models. These supplementary analyses were not included in the statistical analysis plan of the study protocol and are intended to complement the main findings of the trial. Our estimand, the per-protocol effect, falls under the category of “hypothetical strategy” as defined by the ICH E9 addendum framework. All analyses were conducted with SAS version 9.4.

### Sensitivity analyses

We conducted several sensitivity analyses to examine the robustness of our findings. First, to adjust for missing values on the VACS index at 18 months, we replicated the original intention-to-treat analysis using inverse probability censoring weighting rather than multiple imputation. Second, to examine trends by level of adherence, we re-ran the analyses using different thresholds for high adherence: 70% and 90% of the average self-reported pill coverage. Third, we re-ran the analysis defining high adherence based on positive riboflavin tests in urine rather than on self-reported pill counts in the previous 6 weeks. Finally, we refit the inverse probability weight models to adjust for confounders measured at the same visit rather than at the most recent previous visit.

## Results

Of the 248 participants included in this study, 73% were men, 74% reported heavy drinking 7 days prior to baseline, and 37% reported injection drug use 30 days prior to baseline. Their median (interquartile range [IQR]) CD4 cell count, HIV RNA, and age at baseline were 462 [298, 691] cells/mm^3^, 4.43 [3.56, 5.05] log10 copies/mL, and 33 [30, 37] years, respectively (Table 1). Of the included individuals, 64 and 61 remained uncensored until the end of the study (18 months) in the zinc and placebo groups, respectively. The median [IQR] average cumulative adherence at 18 months was 85% [56, 94] and 84% [68, 95] in the zinc and placebo groups, respectively.

**Table 1:** Baseline characteristics of 248 eligible participants in the ZINC trial for HIV disease among alcohol users, 2013–2015

Baseline variable	Placebo group (*N* = 126)	Zinc group (*N* = 122)	All
Gender, *N* (%)			
Male	90 (71%)	90 (74%)	180 (73%)
Female	36 (29%)	32 (26%)	68 (27%)
Depressive symptoms in the previous 7 days, *N* (%)			
Yes	50 (40%)	54 (45%)	104 (42%)
No	75 (60%)	66 (55%)	141 (58%)
Unknown	1 (< 1%)	2 (< 1%)	3 (< 1%)
Heavy drinking in the previous 7 days, *N* (%)			
Yes	93 (74%)	91 (75%)	184 (74%)
No	33 (26%)	31 (25%)	64 (26%)
Injection drug use in the previous 30 days, *N* (%)			
Yes	47 (38%)	44 (36%)	91 (37%)
No	77 (62%)	77 (64%)	154 (63%)
Previous diagnosis of cardiovascular disease, *N* (%)			
Yes	5 (4%)	5 (4%)	10 (4%)
No	121 (96%)	117 (96%)	238 (96%)
Hepatitis C virus infection			
Yes	113 (90%)	105 (86%)	222 (89%)
No	13 (10%)	17 (14%)	26 (11%)
Age in years, median [IQR]	33 [30, 37]	33 [30, 37]	33 [30, 37]
CD4 cell count in cells/mm^3^, median [IQR]	480 [304, 683]	443 [290, 693]	462 [298, 691]
HIV RNA in log10 copies/mL, median [IQR]	4.53 [3.73, 5.02]	4.30 [3.44, 5.12]	4.43 [3.56, 5.05]
VACS index, median [IQR]	24 [18, 31]	24 [18, 31]	24 [17, 35]

*N* number, *IQR* interquartile range, *VACS* Veterans Aging Cohort Study

Table 2 shows the estimated change in the VACS index among individuals with high and low adherence by treatment group. In the placebo group, the estimated mean change between baseline and 18 months was 5.84 (95% confidence interval 0.73, 12.1) under high adherence and 2.70 (− 5.80, 15.06) under low adherence. Corresponding estimates in the zinc group were −2.16 (− 8.07, 3.59) and 5.23 (− 2.19, 10.78). The estimated mean difference in the change between the zinc and placebo groups under high adherence (i.e., the per-protocol effect estimate) was − 8.01 (− 16.42, 0.01) (Table 3). This estimate was somewhat higher than the original intention-to-treat estimate of − 4.68 (− 9.62, 0.25), but the confidence interval still included zero.

**Table 2:** Change in VACS index (95% confidence interval) between baseline and 18 months by randomization group and cumulative adherence (high versus low). Estimated using inverse probability weighting. ZINC trial 2013–2015

Randomization group	High adherence^a^	Low adherence^b^	Difference (high versus low adherence)
Zinc	−2.16 (−8.07, 3.59)	5.23 (−2.19, 10.78)	− 7.39 (−17.13, 2.42)
Placebo	5.84 (0.73, 11.80)	2.70 (−5.80, 15.06)	3.14 (− 9.90, 13.84)

^a^High adherence: cumulative adherence ≥80% based on self-reported pill count

^b^Low adherence: cumulative adherence < 80% based on self-reported pill count

**Table 3:** Change in VACS index between baseline and 18 months by randomization group and cumulative adherence (high versus low) under different methods assumptions. ZINC trial 2013–2015

	Adjusted mean difference in change from baseline to 18 months (95% CI)
Per-protocol effect—main analysis^a^	−8.01 (− 16.42, 0.01)
Per-protocol effect—high adherence ≥70%^a^	−4.07 (− 11.50, 2.75)
Per-protocol effect—high adherence ≥90%^a^	− 12.34 (− 12.14, − 4.14)
Per-protocol effect—high adherence ≥80%—riboflavin test^a^	−3.48 (− 10.44, 4.27)
Per-protocol—change in the temporal order of post-baseline covariates^a^	− 7.63 (− 15.21, 0.39)
Intention-to-treat with inverse probability of censoring weights^a^	−7.08 (− 13.98, 0.04)
Intention-to-treat—original analysis with multiple [4] imputation	− 4.68 (− 9.62, 0.25)
Per-protocol—original “standard” analysis [4]	−7.49 (− 13.74, − 1.23)

^a^Estimated using inverse probability weighting

In the sensitivity analysis in which we examined alternative thresholds for the definition of adherence, the mean difference in the change between individuals in the zinc and placebo groups under high adherence was − 4.07 (− 11.5, 2.75) and − 12.34 (− 20.14, − 4.14) for high adherence defined as 70% and 90% of pill coverage, respectively. The mean difference in change was − 3.48 (− 10.44, 4.27) when high adherence was defined as ≥80% of urine tests being positive for riboflavin.

## Discussion

This study presents the per-protocol effect of zinc supplementation in the ZINC trial under the ideal conditions of high adherence and no loss to follow-up. We used the inverse probability weighting to adjust for baseline and post-randomization confounders. Our per-protocol effect estimate of the mean difference in change in the VACS index between the zinc and placebo groups was − 8.01 (95% CI − 16.42, 0.01), somewhat larger than the intention-to-treat effect difference in change (− 4.68 (− 9.62, 0.25)), but also not statistically significant. This suggests that the intention-to-treat estimate may have underestimated the beneficial effect of zinc supplementation in PLWH who engage in heavy drinking. However, like in the intention-to-treat effect estimate, confidence intervals were wide.

We found that the mean difference in change between individuals in the zinc and placebo groups under high adherence was − 4.07, − 8.01, and − 12.34 for high adherence defined as 70%, 80%, and 90% of pill coverage, respectively. This apparent trend of increasing effect magnitude with increasing thresholds for the definition of high adherence is suggestive of a potential beneficial effect of high adherence to zinc supplementation and requires further exploration through new studies.

The intention-to-treat approach is the gold standard for estimating the causal effect of interventions in randomized controlled trials. However, in double-blinded randomized trials with suboptimal adherence to the study interventions, the intention-to-treat effect might provide biased estimates and should be complemented with per-protocol effect estimates. Unlike the intention-to-treat effect, the per-protocol effect quantifies the maximum benefit of a treatment/intervention, and usually, clinicians and patients making treatment decisions find the per-protocol effect estimates more informative than the intention-to-treat estimates. g-methods have been typically developed and used for the analysis of large observational studies and for the per-protocol analysis of large randomized trials [10, 12]. Our study is a rare example of a per-protocol analysis of a relatively small clinical trial using inverse probability weighting. Because of the relatively small sample size and the large proportion of individuals who were lost to follow-up, the confidence intervals for our estimates were wide. Fully parametric methods, such as the parametric g-formula, may offer an alternative way to estimate the per-protocol effect in a small trial.

Our per-protocol effect estimate was similar to the standard per-protocol estimates, which restricted analyses to individuals who reported high adherence over three visits. This similarity should not be interpreted as an argument in favor of standard per-protocol analysis, because such estimates are generally subject to bias. They rely on the unrealistic assumptions that adherence and loss to follow-up occur completely at random. In contrast to the standard approach, we adjusted our analyses for baseline and post-baseline characteristics likely to influence adherence.

Our analysis has limitations. First, self-reported adherence might be an imperfect measurement of pill uptake. A sensitivity analysis using an alternative measure of adherence based on the riboflavin urine test showed a somewhat smaller benefit of zinc supplementation. This discrepancy may arise from the difference in timing of the two adherence assessments: self-reported adherence was defined as the percentage of pills taken in the previous 6 weeks on a visual analog scale, while the riboflavin test allows detection of medication intake occurring in the previous 24 h. Second, our analyses assume that all prognostic factors that predict adherence are identified and accurately measured. Our estimates would be biased if one or more important determinants of adherence were not included in the model. However, we found no adherence effect in the placebo group. This is reassuring as it indicates that the available data are sufficient to adjust for confounding and selection bias due to loss to follow-up. Third, when adherence was not measured due to a missed refill visit, we carried forward the measure of adherence from the most recent visit and censor follow-up at the first missing visit. This may have led to overestimating adherence, if individuals who did not refill their medications discontinued treatment. Finally, we censored follow-up at the time of death or a missed study visit and used inverse probability of censoring weighting to adjust our estimates for informative censoring. Our estimates would be biased if censoring remains informative conditionally on the baseline and time-varying confounders included in the models for censoring weights.

In conclusion, we used the rich data collected by the ZINC trial to estimate the per-protocol effect of zinc supplementation on the VACS index at 18 months while adjusting for both non-adherence and loss to follow-up. High adherence to zinc was associated with a lower VACS index score, but the confidence intervals were wide. Our per-protocol effect estimation confirms the potential benefits of daily zinc supplementation in PLWH who engage in heavy drinking. Further studies examining larger sample sizes are needed to shed more light on the potential benefits of zinc supplementation in PLWH who engage in heavy drinking in Russia and elsewhere.

## Supplementary Information

**Additional file 1:** Appendix – models. Appendix Figure 1. Timelines of the ZINC trial.

## Data Availability

The datasets used and/or analyzed during the current study are available from the corresponding author on reasonable request.
