# Supplementary material for: Per-protocol analysis of the ZINC trial for HIV disease among alcohol users
Source: Trials. 2021 Mar 23;22:226. doi: 10.1186/s13063-021-05178-9 (PMC7989012; doi:10.1186/s13063-021-05178-9)

**Appendix**

Appendix - models

The following describe the process of estimating the per-protocol effects in the ZINC trial.

1. Inverse probability weighting estimation

We define *Z* as an indicator for randomization group (Z=1 zinc, Z=0 placebo), A_t_ an indicator for high adherence at time *t* (A_t_=1 high adherence, A_t_=0 low adherence), $\bar{A}_{t}$the history of adherence up to time *t*, C_t_ an indicator for loss to follow-up at time *t*, **V** a set of baseline covariates, $\bar{L}_{t}$the covariate history at time *t*. The list of baseline and time-varying covariates is presented in Methods.

Step 1. Fit a pooled logistic regression model for the probability of high adherence conditional on the baseline and time-varying covariates:

Logit (P(A_t_=1|C_t_=0,**V**, ${\bar{\mathbf{L}}}_{t}$, A_t-1_, Z=0)= φ_0_ + φ_1_V+ φ_2_ L_t-1_+ φ_3_ A_t-1_ + φ_3_ f(t)

where f(t) is flexible function of time (we used a quadratic function of month of follow-up)

Step 2. Fit a pooled logistic regression model for the probability of high adherence conditional on baseline covariates and previous adherence only:

Logit (P(A_t_=1|C_t_=0,**V**, A_t-1_, Z=0)= η_0_ + η _1_V+ η _3_ A_t-1_ + η _3_ f(t)

Step 3. Calculate the stabilized inverse probability of adherence weights as

SW^a^_t_=Π_0_^t^ P(A_t_=1|C_t_=0,**V**, A_t-1_, Z=0)/ P(A_t_=1| C_t_=0,**V**, ${\bar{\mathbf{L}}}_{t}$, A_t-1_, Z=0)

using the predicted probabilities of adherence from logistic regression models in Step 1 and 2

Step 4. Repeat this process for the conditional probability of being uncensored at study visits

Logit (P(C_t_=0|C_t-1_=0,**V**, A_t-1_, Z=0)= γ_0_ + γ_1_V+ γ_2_ L_t-1_+ γ_3_ A_t-1_ + γ_4_ f(t)

Logit (P(C_t_=0| C_t-1_=0,**V**, ${\bar{\mathbf{L}}}_{t-1}$, A_t-1_, Z=0)= φ_0_ + φ_1_V+ φ_2_ L_t-1_+ φ_3_ A_t-1_ + φ_3_ f(t)

Step 5. Calculate the stabilized censoring weights as:

SW^C^_t_=Π_0_^t^ P(C_t_=0| C_t-1_=0,**V**, A_t-1_, Z=0)/ P(C_t_=0|C_t-1_=0,**V**, ${\bar{\mathbf{L}}}_{t-1}$, A_t-1_, Z=0)

using the predicted probabilities of being uncensored from logistic regression models in Step 4.

Step 6. Calculate the final inverse probability weights as SW_t_= SW^C^_t_ x SW^A^_t_

We expect these weights to have a mean of 1.

Step 7. Repeat steps 1 to 6 on subset of individuals with Z=1, ie zinc group

1. Outcome model

Let *Y_18_* be an indicator for change in VACS index between baseline and 18 months (end of the study), *cum(*$\bar{A}_{t}$*)* an indicator for high cumulative incidence up to time *t* and *C_18_=0* an indicator for remaining under follow-up until month 18.

Step 1: Fit the following linear regression model on the subset of individuals with *Z=0* (placebo) applying inverse probability weights:

*(Y_18_|C_18_=0, cum(*$\bar{A}_{18}$*),Z=0)= α_0_ + α _1_ cum(*$\bar{A}_{18}$*)*

The predicted change under high cumulative adherence is *α _0_ + α _1_*

The predicted change under low cumulative adherence is *α _0_*

The predicted difference of high versus low adherence is *α _1_*

Step 2: Fit the following linear regression model on the subset of individuals with *Z=1* (zinc) applying inverse probability weights:

*(Y_18_|C_18_=0, cum(*$\bar{A}_{18}$*),Z=1)=* β_0_ *+* β_1_ *cum(*$\bar{A}_{18}$*)*

The predicted change under high cumulative adherence is *β_0_ + β_1_*

The predicted change under low cumulative adherence is *β_0_*

The predicted difference of high versus low adherence is *β_1_*

Step 3: Estimate the per-protocol effect as:

(Y_18_|C_18_=0,cum($\bar{A}_{18}$)=1,Z=1) – (Y_18_|C_18_=0,cum($\bar{A}_{18}$)=1,Z=0) = (α _0_ + α _1_) – (β_0_ + β_1_)

Step 4: We can also adjust the models in Step 1 and Step 2 for the baseline covariates. In our analyses we adjusted for randomization stratification factors (gender and heavy drinking at baseline)

3 Confidence intervals

To estimate confidence intervals we repeat the process, including the estimation of the inverse probability weights and outcome model, using non-parametric bootstrap with 500 samples from the original dataset

Appendix Figure 1. Timelines of the ZINC trial.


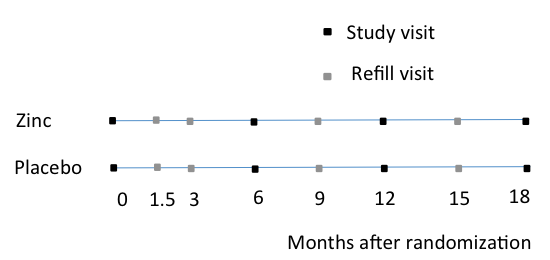

Supplement: Supplementary file 1 — Additional file 1: Appendix – models. Appendix Figure 1. Timelines of the ZINC trial. [file 13063_2021_5178_MOESM1_ESM.docx]
